# An Allele of *Sequoia* Dominantly Enhances a *Trio* Mutant Phenotype to Influence Drosophila Larval Behavior

**DOI:** 10.1371/journal.pone.0084149

**Published:** 2013-12-20

**Authors:** Kathryn E. Dean, April Fields, Marcus J. Geer, Eric C. King, Brian T. Lynch, Rohan R. Manohar, Julianne R. McCall, Katherine C. Palozola, Yan Zhang, Eric C. Liebl

**Affiliations:** Department of Biology, Denison University, Talbot Hall of Biological Science, Granville, Ohio, United States of America; McGill University, Canada

## Abstract

The transition of *Drosophila* third instar larvae from feeding, photo-phobic foragers to non-feeding, photo-neutral wanderers is a classic behavioral switch that precedes pupariation. The neuronal network responsible for this behavior has recently begun to be defined. Previous genetic analyses have identified signaling components for food and light sensory inputs and neuropeptide hormonal outputs as being critical for the forager to wanderer transition. Trio is a Rho-Guanine Nucleotide Exchange Factor integrated into a variety of signaling networks including those governing axon pathfinding in early development. Sequoia is a pan-neuronally expressed zinc-finger transcription factor that governs dendrite and axon outgrowth. Using pre-pupal lethality as an endpoint, we have screened for dominant second-site enhancers of a weakly lethal *trio* mutant background. In these screens, an allele of *sequoia* has been identified. While these mutants have no obvious disruption of embryonic central nervous system architecture and survive to third instar larvae similar to controls, they retain forager behavior and thus fail to pupariate at high frequency.

## Introduction

Connecting specific neural networks to specific behaviors or skills is a long-term goal of neurobiology. Toward that end model systems such as Drosophila have been useful, allowing specific neural structures as well as specific gene products to be linked to complex behaviors such as courtship and mating [1]–[3]. Another classic behavior that has been well studied in Drosophila is the foraging behavior of third instar larvae. As third instar larvae prepare to pupate they switch from being foragers, which are focused on feeding and are photo-phobic, to wanderers, which are not focused on feeding and are photo-neutral [4]–[7]. Several gene products have been implicated in the behavioral switch from foragers to wanderers; these include neuropeptides [5], enzymes involved in neuropeptide metabolism [8], ion channels [9], a mitochondrial DNA polymerase [10], and a scaffolding protein [11].

Identifying dominant second-site enhancers of a sensitized genetic background via modifier screens is a powerful technique to dissect signaling networks [12]. For example, the Abl tyrosine kinase is a regulator of axon outgrowth [13], and such second-site modifier screens have identified numerous dominant enhancers of the *abl* mutant phenotype with strong phenotypic effects on central nervous system (CNS) architecture [14]–[17]. Trio is a neuronally expressed Rho-Guanine Nucleotide Exchange Factor (GEF) involved in axon pathfinding during early embryogenesis [14], [18]–[20]. We have used second-site modifier genetics to explore signaling networks that involve Trio. Specifically, we have screened for mutations that dominantly exacerbate the pre-pupal lethality of a weakly lethal *trio* mutant background. In these screens we have identified an allele of the zinc-finger transcription factor *sequoia*. However, rather than grossly affecting axon pathfinding during embryogenesis, this genetic combination inhibits the behavioral switch from foragers to wanderers during the third larval instar.

## Materials and Methods

### Fly stocks and maintenance

All flies were maintained on either standard cornmeal-yeast medium or Formula 4–24 Instant Food (Carolina Biological) in humidified incubators. All fly stocks were obtained from the Bloomington Stock Center except for the *trio^M89^* stock that was generated in our laboratory and has been described previously [14].

### Mutagenesis and screening for dominant enhancers of the *trio* pre-pupal lethality

Males with wild-type second chromosomes and third chromosomes carrying the *trio^M89^* mutation were subjected to 45 Gray of gamma radiation at the Ohio State University Nuclear Reactor Laboratory. Mutagenized second and third chromosomes were captured over the *T(2;3) SM6a-TM6B* balancer chromosomes which is marked with the dominant phenotypic markers *Tubby* (*Tb)*, and *Curly* (*Cy*). The Tubby phenotype is easily scored in pupae. The Curly phenotype is easily scored in adults. Single males were crossed to *+; trio^s036810^/T(2;3) SM6a-TM6B* virgin females. Such crosses should produce a Mendelian ratio of 2 Tubby, Curly : 1 non-Tubby, non-Curly (i.e. wild type) animals. Critically, the non-Tubby, non-Curly offspring represent the *trio* mutant (*trio^s036810^/trio^M89^*) class. Progeny were visually screened for synthetic lethality by scoring for a strong reduction in the frequency of these non-Tubby, non-Curly *trio* mutant (*trio^s036810^/trio^M89^*) pupae and adults. Reductions were quantified by dividing the number of non-Tubby (or non-Curly) animals by half the number of Tubby (or Curly) animals to obtain the “percent expected”. Chromosomes showing less than 25% of expected *trio* mutant pupae were recovered, retested and maintained as balanced stocks.

### Quantifying larvae

The *Tubby* phenotypic marker was used to score larvae. Crosses were carried out on 4–24 Instant food (Carolina Biological) and progeny larvae were recovered by floatation after re-suspending the food plug in 3 M NaCl. “Percent expected” was calculated as described above.

### Mapping and cloning the *9.17* mutation

The *9.17*-containing second chromosome was recovered in unbalanced females over the *wg^Sp-1^, Bl^1^, L^rm^, Bc^1^, Pu^2^* mapping chromosome and multiple (>15) recombinants across each phenotypically scorable interval were recovered and tested for the presence of the *9.17* mutation by assaying for its enhancer activity.

Genomic libraries were made with the CopyControl Fosmid Library Production kit (Epicenter Biotechnologies). Libraries were screened with DIG-probes using standard protocols. All sequencing was done at the Ohio State Plant-Microbe Genomics Facility. Contigs were aligned using Sequencher (Gene Codes Corporation), and BLAST alignments through FlyBase (www.flybase.org).

### Antibody staining of embryos

Antibody staining was carried out on 0–24 hr embryos that were collected, fixed and stained as described previously [14]. Monoclonal antibody BP102 was obtained from the Developmental Studies Hybridoma Bank (University of Iowa, Iowa City, IA). Mouse monoclonal anti-beta-galactosidase antibody (Promega, Madison WI) was used to detect *lacz* expression from enhancer trap-containing balancer chromosomes to distinguish embryos' genotypes.

### Food dispersal assays

Food dispersal assays were carried out similar to those described in Moncalvo and Campos [6]. Synchronized larvae were generated by crossing animals of the appropriate genotype in large cages (Genesee Scientific). Embryos were collected in 2-hour windows on 1.8% agar plates. These plates were removed from the cages and aged for an additional 24 hours at 25°C after which all hatched larvae were removed with a mild suction apparatus. The cleared plates were returned to the 25°C incubator for 3 hours, after which all hatched first instar larvae (age  = 0–3 hours after hatching) were removed and transferred to 100 mm dishes containing 40 ml of 4–24 instant food (Carolina Biological). Larvae were seeded at the defined density of 100 larvae per plate. These synchronized first instar larvae were then aged at 25°C to the appropriate stage (63–66 hours after hatching for young third instar; 90–93 hours after hatching for older third instar) and isolated by re-suspending the food in 3 M NaCl. The buoyant, floating larvae were skimmed off the surface, washed with distilled water and starved for 60 minutes at 25°C on moist filter paper. Animals were then transferred to the middle of a 25 mm×10 mm plug of 4–24 instant food placed on a 3% agar base in a 100 mm petri dish. After 90 minutes at 25°C, the food plug was scooped up and larvae within recovered by floatation with 3 M NaCl. Larvae that had migrated off the food plug were recovered with a paintbrush. Larvae were rinsed, transferred to 95% ethanol and phenotypically classified using the *Tubby* marker.

### Larval crawling assays

Larval crawling assays were carried out similar to those described in Nichols *et al.*, [21]. Synchronized larvae were generated as described above. Individual larvae were then transferred to 100 mm dishes with 2% agar over 2 mm grid paper. The number of lines the larva's mouth crossed in 60 seconds was recorded.

### Larval mass determination

Synchronized larvae were generated as described above. The mass of individual larvae was determined by duplicate measurements with an Ohaus Discovery DV215CD balance, accurate to 0.01 mg.

### Larval instar determination

The mouth hooks from individual larvae were dissected under 80% glycerol and examined with 400× magnification. The overall morphology was compared to that shown in Strasburger [22] as referenced in Ashburner [23], and the number of small teeth on the front part of the armature were counted [24].

### Statistical analysis

Data was analyzed using the JMP 10 statistical software package (SAS Institute) and consisted of Chi-squared tests, t-tests, one-way ANOVAs and Tukey-Kramer HSD pairwise analyses.

## Results

### Isolation of the *9.17* mutation as a dominant enhancer of the *trio* pre-pupal lethality

*trio^s036810^* is a hypomorphic allele of the Trio Rho-GEF generated by P-element mutagenesis [14], [18], [20]. *trio^M89^* is an EMS-induced point mutation (L1412F) that disrupts a conserved residue in Trio's first GEF-domain [14]. Animals of the genotype *trio^s036810^/trio^M89^* constitute a genetic background sensitized for Trio activity, as less than expected but still a significant number of these animals survive to both the pupae and adult stages [14] (**Table 1**). In this sensitized background, gamma ray-mutagenized second and third chromosomes were screened for their ability to dominantly exacerbate *trio's* pre-pupal lethality. The *9.17* mutation was isolated in this screen as animals heterozygous mutant for *9.17* and homozygous mutant for *trio* (*9.17/+*; *trio^s036810^/trio^M89^*) demonstrated only 23.9% expected survival to pupae and 5.6% expected survival to adults (**Table 1**). *9.17's* dominant effect was dependent on the *trio* mutant background, as animals heterozygous mutant for *9.17* and heterozygous mutant for *trio* showed robust viability (**Table 1**).

**Table 1:** Dosage-sensitive modification of the *trio* mutant phenotypea.

Genotype	% expected pupae	% expected adults
*+/+; trio^s036810^/trio^M89^*	67.9	67.4
*seq^9.17^/+; trio^s036810^/trio^M89^*	23.9	5.6
*seq^9.17^/+; trio^+^/trio^M89^*	93.0	93.3
*seq^vr5-5^/+; trio^s036810^/trio^M89^*	46.0	38.7
*seq^vr5-48^/+; trio^s036810^/trio^M89^*	50.7	39.5
*Df(2R)BSC273/+; trio^s036810^/trio^M89^*	51.8	35.3

a n = 5 trials with at least 150 animals scored in each trial

### Molecular characterization of the *9.17* mutation

Mutation *9.17* was mapped to the second chromosome. Meiotic recombinational mapping that followed *9.17's* dominant enhancement of the *trio* mutant phenotype placed *9.17* at 64 cM. Scanning across this region with overlapping chromosomal deficiencies uncovered a recessive lethal lesion on the *9.17* chromosome in this exact area (between bases 9,047,084 and 9,106,852 based on the failure of the *9.17* chromosome to complement both *Df(2R)Exel8057* and *Df(2R)BSC273)* [25], [26]. Genes likely to be involved in axon pathfinding and/or Rho family-dependent signal transduction or such genes' potential cis-regulatory sequences that lie within this region include *Fak-like tyrosine kinase* (*PR2*), a Rac-dependent tyrosine kinase [27], *Neurospecific receptor kinase* (*Nrk*), a transmembrane receptor tyrosine kinase expressed on neurons [28] and *sequoia* (*seq*), a zinc-finger transcription factor involved in regulating axon and dendrite morphology [29].

**Figure 1:**
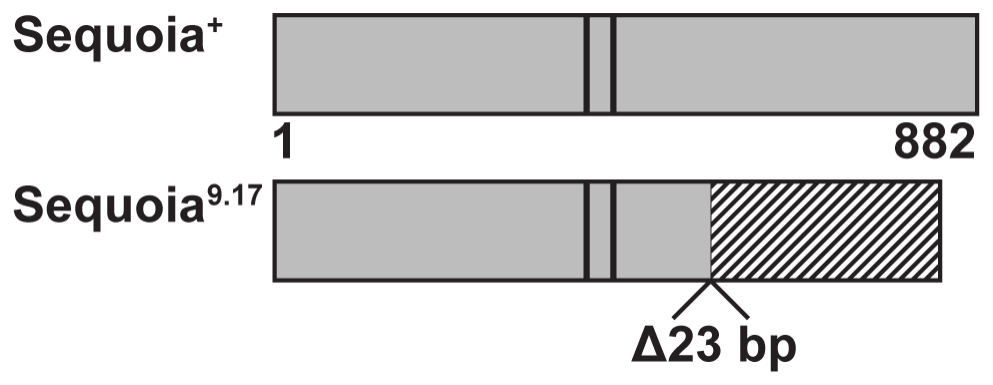
Schematic structures of wild type Sequoia and Sequoia^9.17^. Sequoia^+^ is 882 residues long and contains two C2H2 zinc fingers (D375 to K476) shown as dark vertical lines. The 23 base pair deletion in *seq^9.17^* causes a frameshift after Q561, resulting in 260 unique residues (striped box). Sequoia^9.17^ retains the two CH2H2 zinc fingers.

A fosmid-based genomic library was generated from the balanced *9.17* stock and clones from the *9.17* chromosome within this interval were distinguished from clones from the balancer chromosome based on diagnostic SNPs. From these clones candidate genes' (*PR2, Nrk, seq*) exons and intron/exon boundaries were fully sequenced. All sequences were wild type except for a 23 base-pair deletion that was discovered within *sequoia's* second coding exon. Conceptual translation showed this deletion would cause a frameshift resulting in 260 novel amino acids following Q561, but leaving Sequoia's zinc fingers intact (**Fig. 1**). Parallel cloning and sequencing of *9.17's* parental chromosome showed the *sequoia* gene to be wild type. Thus as 1) this small deletion co-mapped with *9.17's* dominant enhancement of the *trio* mutant phenotype, 2) a 23 base pair deletion is consistent with the gamma ray mutagenesis used to create *9.17*, and 3) the lesion is absent from the parental chromosome, we have identified the *9.17* strong dominant enhancer of the *trio* pre-pupal lethality as *seq^9.17^*.

### Genetic characterization of the *seq^9.17^* allele

A chromosomal deletion that removes *seq* (*Df(2R)BSC273* [25]) and the point alleles *seq^vr5-5^* (Q641STOP[29]) and *seq^vr5-48^* [30] all failed to complement *seq^9.17^* in the *trio^+^* background as judged by adult viability; crossing these *seq* alleles balanced over the *CyO* balancer chromosome with each other gave no straight-winged offspring. However, neither *Df(2R)BSC273, seq^vr5-5^*, nor *seq^vr5-48^* behaved as a strong dominant enhancer of the *trio* pre-pupal lethailty, with approximately 50% of the expected pupae developing in all cases (**Table 1**). Thus while heterozygosity for a *seq* null deletion or for the recessive lethal *seq* alleles *seq^vr5-5^* or *seq^vr5-48^*, in the *trio^s036810^/trio^M89^* mutant background, reduced pupal and adult viability as compared to controls, this enhancement is much less than that seen with *seq^9.17^* (**Table 1**). Therefore *seq^9.17^* behaves differently than all other *seq* alleles tested.

### Phenotypic characterization of *seq^9.17^*'s dominant enhancement of the *trio* pre-pupal lethality

The dominant enhancement of sensitized genetic backgrounds involving genes coding for proteins within axon pathfinding signaling networks often results in strong embryonic CNS phenotypes (e.g. [14]–[17]). However, the embryonic CNS of animals heterozygous mutant for *seq^9.17^* and homozygous mutant for *trio* (*seq^9.17^/+; trio^s036810^/trio^M89^*) was indistinguishable from simple *trio* mutants (*trio^s036810^/trio^M89^*) when visualized with mAb BP102 (x_1_^2^ = 1.01, p = 0.48; **Table 2**). As *seq^9.17^* was identified by its ability to dominantly reduce pupation of *trio* mutant animals, yet those animals had structurally normal embryonic CNSs, we then examined the survival of those animals to larval stages. While survival to pupae was dramatically reduced by the presence of the *seq^9.17^* mutation (67.9% of expected for *trio^s036810^/trio^M89^* vs. 23.9% of expected for *seq^9.17^/+; trio^s036810^/trio^M89^*; x_1_^2^ = 85.29, p<0.0001; **Table 2**), survival to third instar larvae was indistinguishable between the two genotypes (75.5% of expected vs. 67.6% of expected respectively; x_1_^2^ = 1.71, p = 0.19; **Table 2**). Even after 10 days at 25°C, no *seq^9.17^/+; trio^s036810^/trio^M89^* pupae were found “hidden” within or under the food plug; these larvae are retarded in their advancement to pupae. In an effort to understand this reduced ability of *seq^9.17^/+; trio^s036810^/trio^M89^* larvae to pupate, we examined several aspects of these larvae.

**Table 2:** Characterizing the lethal phase of the *seq^9.17^* dominant enhancement.

Life stage	Test	Genotype	Result
Embryos	% segments with disrupted CNS^a^	Simple *trio* mutant^c^	1.4% (n = 147 segments)
		Enhanced *trio* mutant^d^	1.9% (n = 105 segments)
Larvae	% expected recovered^b^	Simple *trio* mutant^c^	75.5%
		Enhanced *trio* mutant^d^	67.6%
Pupae	% expected recovered^b^	Simple *trio* mutant^c^	67.9%
		Enhanced *trio* mutant^d^	23.9%***
Adults	% expected recovered^b^	Simple *trio* mutant^c^	67.4%
		Enhanced *trio* mutant^d^	5.6%***

a Determined by mAb BP102 staining

b n = 5 trials with at least 150 animals scored in each trial

c *+/+; trio^s036810^/trio^M89^*

d *seq^9.17^/+; trio^s036810^/trio^M89^*

***p<0.0001 determined by x^2^

**Figure 2:**
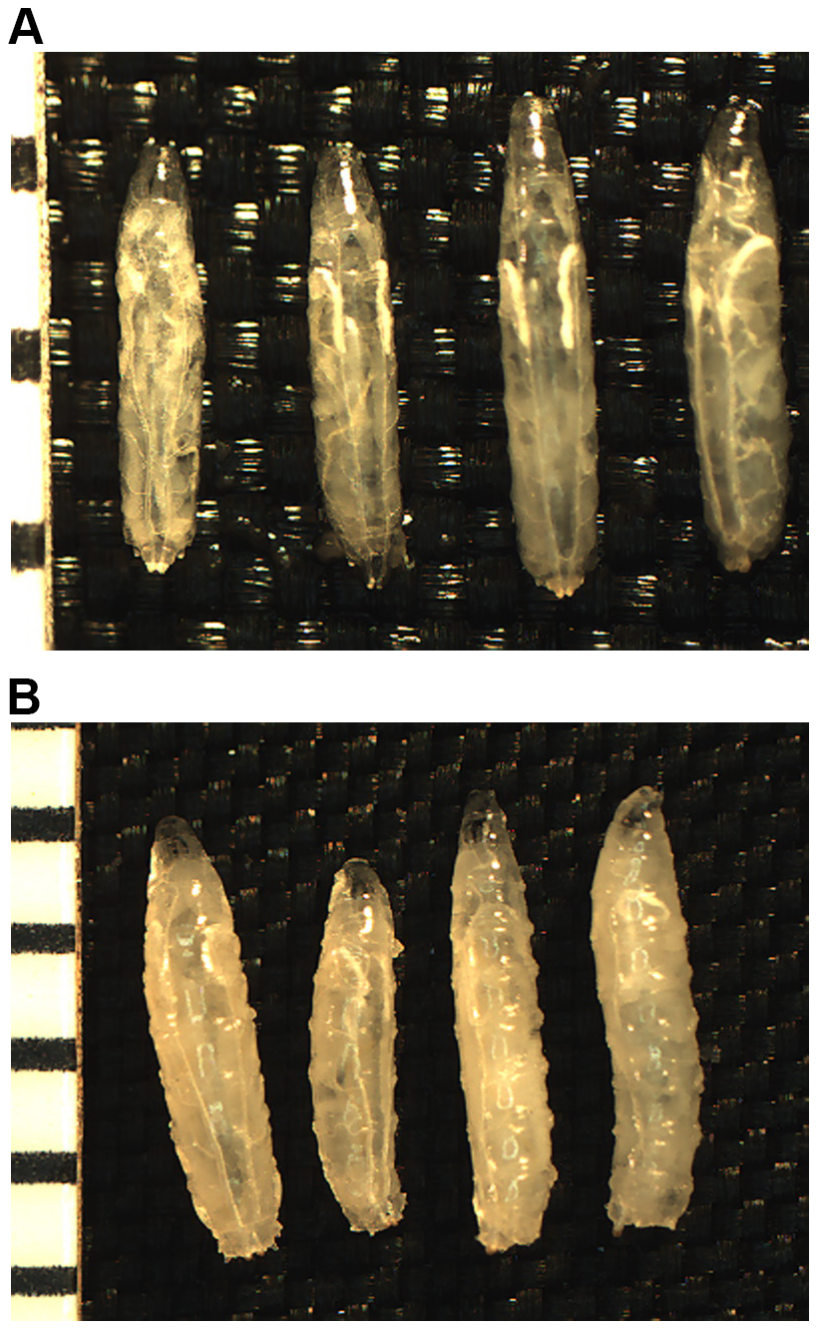
Representative third instar larvae used in phenotypic characterizations. **A**: 63–66 hours after hatching (forager) larvae. The two animals on the left are *seq^9.17^/+; trio^s036810^/trio^M89^*. The two animals on the right are *+/+; trio^s036810^/trio^M89^*. Anterior is up. A millimeter ruler is along the left for scale. **B**: 90–93 hours after hatching (wanderer) larvae. The two animals on the left are *seq^9.17^/+; trio^s036810^/trio^M89^*. The smaller animal is representative of 15% of this population. The two animals on the right are *+/+; trio^s036810^/trio^M89^*. Anterior is up. A millimeter ruler is along the left for scale.

The general locomotive ability, as determined by larval crawling assays, was similar between *+/+; trio^s036810^/trio^M89^* and *seq^9.17^/+; trio^s036810^/trio^M89^* for both young third instar larvae (63–66 hours after hatching; t_28.8_ = −1.64, p = 0.11; **Table 3**) and old third instar larvae (90–93 hours after hatching; t_43.5_ = −1.54, p = 0.13; **Table 4**). The average mass of these young third instar larvae was similar (t_25.8_ = −1.62, p = 0.12; **Table 3**), but the old (90–93 hours after hatching) *seq^9.17^/+; trio^s036810^/trio^M89^* larvae were significantly less massive as compared to control *+/+; trio^s036810^/trio^M89^* larvae (t_42.8_ = −3.58, p = 0.0009; **Table 4**). This reduction in average mass was primarily due to 15% of the *seq^9.17^/+; trio^s036810^/trio^M89^* old (90–93 hours after hatching) larvae being noticeably smaller; representative animals are shown in **Fig. 2**. To confirm that these smaller old (90–93 hours after hatching) larvae were third instars, mouth hooks were examined from a representative sample. The mouth hooks' overall morphology (**Figure S1**) and tooth count on the front part of the armature (between 11 and 12 teeth) confirmed these were third instars [22]–[24].

**Table 3:** Characterizing the *seq^9.17^* dominant enhancement in 63–66 hours after hatching larvae (foragers).

Assay	Units	Larval genotype	Result+/−SEM (n)
General mobility	Lines crossed in 60 sec	Simple *trio* mutant^a^	19.4 +/− 1.2 (18)
		Enhanced *trio* mutant^b^	17.0 +/− 0.8 (15)
Larvae mass	mg per animal	Simple *trio* mutant^a^	0.32 +/− 0.02 (18)
		Enhanced *trio* mutant^b^	0.28 +/− 0.01 (15)
Food dispersal	% remaining in food	Simple *trio* mutant^a^	100 +/− 0.0 (4 trials)
		Simple *trio* mutants' sibs^c^	95.3 +/− 1.9 (4 trials)
		Enhanced *trio* mutant^b^	100 +/− 0.0 (4 trials)
		Enhanced *trio* mutants' sibs^c^	97.8 +/− 0.9 (4 trials)

a +/+; trio^s036810^/trio^M89^

b seq^9.17^/+; trio^s036810^/trio^M89^

c heterozygous mutant for trio; inherited the balancer chromosome carrying the dominant Tubby marker

**Table 4:** Characterizing the *seq^9.17^* dominant enhancement in 90–93 hours after hatching larvae (wanderers).

Assay	Units	Larval genotype	Result +/− SE (n)
General mobility	Lines crossed in 60 sec	Simple *trio* mutant^a^	21.6 +/− 0.9 (21)
		Enhanced *trio* mutant^b^	19.6 +/− 0.9 (25)
Larval mass	mg per animal	Simple *trio* mutant^a^	1.24 +/− 0.05 (21)
		Enhanced *trio* mutant^b^	0.98 +/− 0.05*** (25)
Food dispersal	% remaining in food	Simple *trio* mutant^a^	41.5 +/− 6.1^d^ (4 trials)
		Simple *trio* mutant's sibs^c^	44.8 +/− 9.5^d^ (4 trials)
		Enhanced *trio* mutant^b^	92.5+/− 9.6^e^ (4 trials)
		Enhanced *trio* mutant's sibs^c^	35.8 +/− 2.3^d^ (4 trials)

a +/+; trio^s036810^/trio^M89^

b seq^9.17^/+; trio^s036810^/trio^M89^

c heterozygous mutant for trio; inherited the balancer chromosome carrying the dominant Tubby marker

*** p = 0.0009

d,e Means from food dispersal assays not sharing the same letter were significantly different at p<0.001

While a general “failure to thrive” may contribute to a minority of the *seq^9.17^/+; trio^s036810^/trio^M89^* larvae's inability to pupate, it alone cannot account for the dramatic reduction in this genotype's pupae. Thus we hypothesized that the behavioral switch from foragers to wanderers may be defective in *seq^9.17^/+; trio^s036810^/trio^M89^* larvae, further reducing their representation as pupae. In food dispersion tests, larvae of defined ages reared at defined densities were placed onto food plugs and tested for their preference to stay and feed or disperse from the plug over a 90-minute test period. All progeny of either control crosses (*+; trio^M89^*/*T(2;3) SM6a-TM6B X +; trio^s036810^/T(2;3) SM6a-TM6B*) or experimental crosses (*seq^9.17^; trio^M89^*/*T(2;3) SM6a-TM6B X +; trio^s036810^/T(2;3) SM6a-TM6B*) were tested together in one arena, and so the *trio* heterozygous mutant siblings (the phenotypically Tubby larvae) served as an internal control against which the *trio* homozygous mutant siblings (the phenotypically non-Tubby, or wild type larvae) were compared.

Young (63–66 hours after hatching) third instar larvae of all genotypes showed typical forager behavior with 95 to 100% of the animals staying within the food plug (**Table 3**). A majority of the old (90–93 hours after hatching) third instar larvae from control crosses showed typical wanderer behavior. Only 41.5% of the *trio^s036810^/trio^M89^* larvae stayed within the food plug, as did 44.8% of their siblings that had inherited a balancer chromosome, and thus were simply heterozygous mutant for *trio* (**Table 4**). A majority of the old (90–93 hours after hatching) third instar larvae from experimental crosses that had inherited a balancer chromosome and thus were simply heterozygous mutant for *trio* (half of which were also heterozygous mutant for *seq^9.17^*) showed typical wanderer behavior in the same assays, with only 35.8% staying within the food plug (**Table 4**). In contrast, most old (90–93 hours after hatching) *seq^9.17^/+; trio^s036810^/trio^M89^* third instar larvae retained typical forager behavior, with 92.5% of the animals staying within the food plug (F_3,12_ = 17.62, p = 0.0001; **Table 4**). Thus the *seq^9.17^/+; trio^s036810^/trio^M89^* larvae retained the forager-like feeding behavior at a time (90–93 hours after hatching) when their siblings and all control larvae had transitioned to wanderers.

## Discussion

By assaying for a clear reduction in pupariation, this work has isolated a strong dominant enhancer of the *trio* pre-pupal lethality (**Table 1**). That enhancer has been identified as a unique allele of the zinc-finger transcription factor Sequoia (**Fig. 1**). The phenotypic basis of this dosage-sensitive genetic interaction has been characterized as a disruption in normal third instar larval growth and behavior. Fifteen percent of the *seq^9.17^/+; trio^s036810^/trio^M89^* old (90–93 hours after hatching) larvae are noticeably smaller, and the average mass of larvae of this age and genotype is statistically lower than controls (**Fig. 2** **,** **Table 4**). Additionally, *sequoia* heterozygous, *trio* homozygous mutant animals retain forager-like behavior (**Table 4**). The failure of these larvae to transition to the wanderer stage is likely a strong contributor to their failure to appear as pupae.

The successful transition of third instar larvae from foragers to wanderers must require the successful integration of a variety of external sensory cues and internal hormonal signals. Multiple mutations that interfere with the transition from foragers to wanderers disrupt various aspects of that general signaling network. Inputs from the environment sensed by larvae include food and light. Pickpocket1 (PPK1), a degenerin/epithelial sodium channel subunit, is expressed in class IV multiple dendritic neurons that completely tile the larval body wall [31]. Mutations in *PPK1* disrupt responses to food-associated stimuli required during the early third instar and therefor disrupt the forager to wanderer transition [9]. Mutations in *tamas* (*tam*) were identified by their disruption of normal responses of foraging third instar larvae to light, and *tam* mutants disrupt larval and adult visual system development [10]. *tam* encodes the mitochondrial DNA polymerase catalytic subunit DNApoly-gamma125. The transition from photo-phobic foragers to photo-neutral wanderers has been linked to serotonin signaling in the brain. Reduced serotonin signaling or targeted inactivation of serotonergic neurons increases late third instar larval responses to light [6].

Neuropeptide signals within the CNS have also been identified as essential for the forager to wanderer transition. Mutations in *amontillado* (*amon*), which codes for a prohormone processing protease, fail to exhibit wanderer behavior [32], and *amon* mutant animals have impaired production of a variety of bioactive neuropeptide hormones [8]. Mutations in *neuropeptide F* (*npf*), the Drosophila homolog of mammalian neuropeptide Y, result in premature transition to wanderer behavior, demonstrating the importance of NPF in food attraction behavior [5]. Indeed, *npf* is normally silenced during the transition to wanderers and inappropriately extended production of NPF inhibits the transition to wanderers [5]. Mutations in the RanBPM scaffolding protein cause early wanderer behavior, although the molecular basis for this is unclear. Significantly, RanBPM is highly expressed in the larval Mushroom Body Kenyon cells, and the mutant phenotype is rescued by targeted expression of wild-type RanBPM to the Kenyon neurons [11], linking that specific larval CNS structure to this behavior.

All of the genetic effects on the forager to wanderer transition summarized above are due to simple homozygous mutations. The present work is unique in that its disruption of normal larval behavior is due to a dosage-sensitive genetic interaction between *trio* and *sequoia* alleles; both the simple *trio* mutant animals and the *sequoia* heterozygoes on their own show normal behavior (**Table 1** **,** **Table 4**), and neither gene has been previously linked to third instar larval behaviors. Dosage-sensitive genetic interactions often indicate close mechanistic, functional interactions between the two gene products or processes controlled by them (e.g. [33]–[36]).

What structure or activity might underlie the dosage-sensitive interaction between *trio* and *sequoia* that disrupts the forager to wanderer transition? Both genes are broadly expressed throughout the CNS and both broadly govern axon and/or dendrite outgrowth and morphology [29], [37]. Therefore, these larvae may have disruptions in neuronal pathways facilitating critical inputs (such as food or light signals), pathways facilitating outputs such as neuropeptide signals, or pathways governing the integration of these inputs and outputs. One convergence between previously identified work on the forager to wanderer transition and *trio* is the larval Mushroom Body. *trio* is strongly expressed in most third instar larval Mushroom Body structures, and *trio* mutant animals have severe, specific defects in the larval vertical and larval medial Mushroom Body lobes [18]. *trio* has been further linked to adult Mushroom Body development through dosage-sensitive genetic interactions with the L1-type CAM Neuroglian (Nrg), strongly suggesting Trio is a downstream effector of Nrg signaling [38]. As the larval Mushroom Body develops as a series of complexly choreographed waves of axon projections [39], it is not unexpected that Trio plays a critical role in its development. However, while *seq* is reportedly expressed at moderate levels in the larval CNS [40], this has not been specifically reported for the larval Mushroom Body. Thus the physical basis of the dosage-sensitive interaction between *trio* and *sequoia* remains an active area of investigation in our laboratory.

While this work has focused on the failure of *seq^9.17^/+; trio^s036810^/trio^M89^* larvae to transition from third instar larvae to pupae, the animals that do develop to pupae then fail to eclose as adults at high rates (**Table 2**). Therefore, the dosage-sensitive genetic interaction between *trio* and *sequoia* likely extends to structures and/or processes critical for eclosion.

A long-term goal of neurobiology is to connect specific behaviors to specific genes' activities and specific neural structures. This work has identified two genes, previously tied to embryonic dendrite and axon development, as having a dosage-sensitive genetic interaction impacting a classic larval behavior. Ongoing studies are directed at testing whether this genetic interaction has a structural basis within the central nervous system.

## Supporting Information

Figure S1 **Representative mouth hooks from 90–93 hours after hatching (wanderer),** ***seq^9.17^/+; trio^s036810^/trio^M89^*** **, third instar larvae. A:** Mouth hook from a normal-sized larvae, representing 85% of the population, viewed under 400× magnification. Morphology and tooth count are consistent with third instar larvae. **B:** Mouth hook from a smaller-sized larvae, representing 15% of the population, viewed under 400× magnification. Morphology and tooth count are consistent with third instar larvae.
(TIFF)
