# Supplementary figures and images for: An Allele of Sequoia Dominantly Enhances a Trio Mutant Phenotype to Influence Drosophila Larval Behavior
Source: PLoS One. 2013 Dec 20;8(12):e84149. doi: 10.1371/journal.pone.0084149 (PMC3869853; doi:10.1371/journal.pone.0084149)

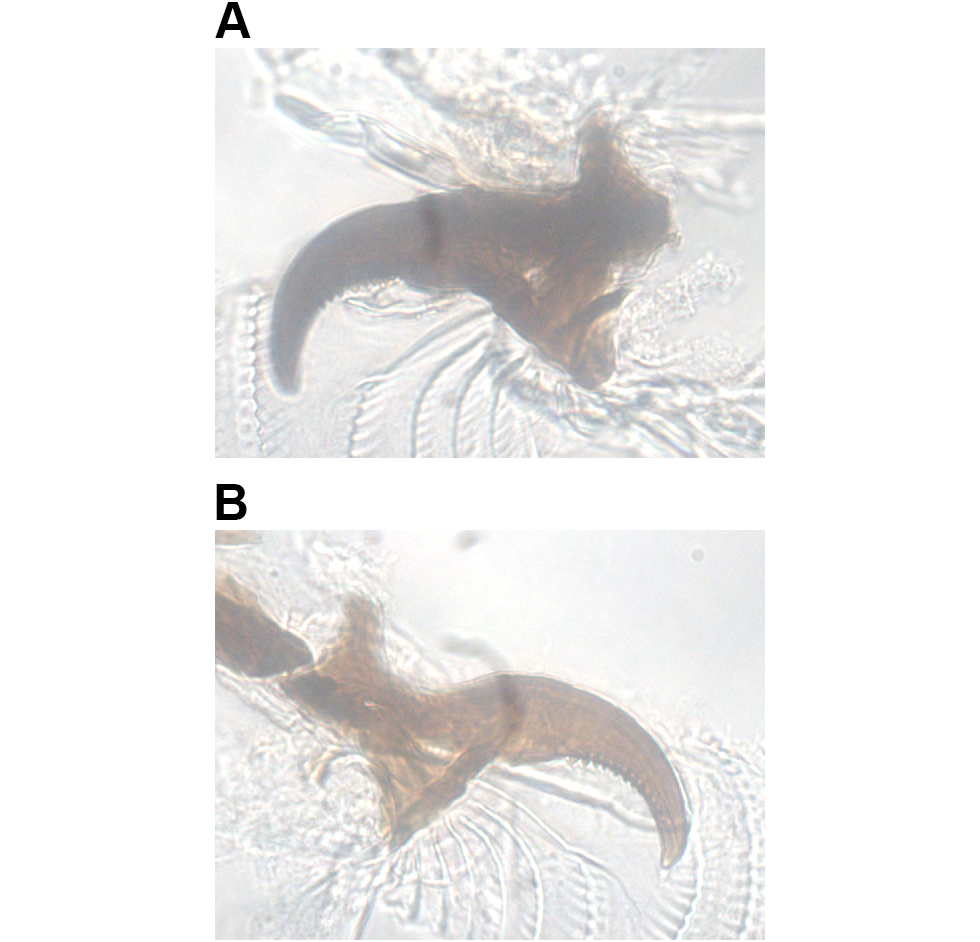

Supplement: Figure S1 — Representative mouth hooks from 90–93 hours after hatching (wanderer), seq9.17/+; trios036810/trioM89 , third instar larvae. A: Mouth hook from a normal-sized larvae, representing 85% of the population, viewed under 400× magnification. Morphology and tooth count are consistent with third instar larvae. B: Mouth hook from a smaller-sized larvae, representing 15% of the population, viewed under 400× magnification. Morphology and tooth count are consistent with third instar larvae. (TIFF) [file pone.0084149.s001.tiff]
